# 3-(Adamantan-1-yl)-1-[(4-ethyl­piperazin-1-yl)meth­yl]-4-[(*E*)-(4-hy­droxy­benzyl­idene)amino]-1*H*-1,2,4-triazole-5(4*H*)-thione

**DOI:** 10.1107/S1600536812005107

**Published:** 2012-02-10

**Authors:** Ali A. El-Emam, Khalid A. Alrashood, Abdul-Malek S. Al-Tamimi, Seik Weng Ng, Edward R. T. Tiekink

**Affiliations:** a Department of Pharmaceutical Chemistry, College of Pharmacy, King Saud University, Riyadh 11451, Saudi Arabia; b Department of Chemistry, University of Malaya, 50603 Kuala Lumpur, Malaysia; c Chemistry Department, Faculty of Science, King Abdulaziz University, PO Box 80203 Jeddah, Saudi Arabia

## Abstract

In the title thione, C_26_H_36_N_6_OS, the 1,2,4-triazole ring is planar (r.m.s. deviation = 0.020 Å) and the benzene ring is twisted out of this plane [dihedral angle = 62.35 (12)°]. Supra­molecular zigzag chains feature in the crystal packing. These are sustained by O—H⋯N(piperazine) hydrogen bonds, and are connected into the three-dimensional crystal structure by C—H⋯S and C—H⋯O inter­actions. The crystal studied was a racemic twin.

## Related literature

For the biological activity of adamantyl derivatives, see: Vernier *et al.* (1969 ▶); El-Emam *et al.* (2004 ▶); Kadi *et al.* (2007 ▶, 2010 ▶); Al-Omar *et al.* (2010 ▶). For related adamantane structures, see: Al-Tamimi *et al.* (2010 ▶); Kadi *et al.* (2011 ▶).
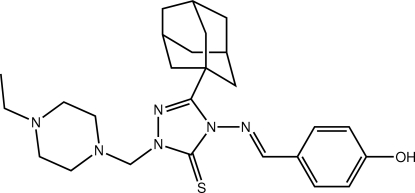


## Experimental

### 

#### Crystal data

C_26_H_36_N_6_OS
*M* *_r_* = 480.67
Monoclinic, 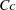

*a* = 16.1006 (6) Å
*b* = 14.2182 (5) Å
*c* = 11.4641 (4) Å
β = 92.440 (4)°
*V* = 2622.00 (16) Å^3^
*Z* = 4
Mo *K*α radiation
μ = 0.15 mm^−1^
*T* = 100 K
0.25 × 0.20 × 0.15 mm

#### Data collection

Agilent SuperNova Dual diffractometer with Atlas detector
Absorption correction: multi-scan (*CrysAlis PRO*; Agilent, 2011 ▶) *T*_min_ = 0.963, *T*_max_ = 0.977
12526 measured reflections
4840 independent reflections
4374 reflections with *I* > 2σ(*I*)
*R*_int_ = 0.036

#### Refinement

*R*[*F* ^2^ > 2σ(*F* ^2^)] = 0.039
*wR*(*F* ^2^) = 0.100
*S* = 1.03
4840 reflections
312 parameters
3 restraints
H atoms treated by a mixture of independent and constrained refinement
Δρ_max_ = 0.25 e Å^−3^
Δρ_min_ = −0.18 e Å^−3^
Absolute structure: Flack (1983 ▶), with 1811 Friedel pairs
Flack parameter: 0.06 (7)

### 

Data collection: *CrysAlis PRO* (Agilent, 2011 ▶); cell refinement: *CrysAlis PRO*; data reduction: *CrysAlis PRO*; program(s) used to solve structure: *SHELXS97* (Sheldrick, 2008 ▶); program(s) used to refine structure: *SHELXL97* (Sheldrick, 2008 ▶); molecular graphics: *ORTEP-3* (Farrugia, 1997 ▶) and *DIAMOND* (Brandenburg, 2006 ▶); software used to prepare material for publication: *publCIF* (Westrip, 2010 ▶).

## Supplementary Material

Crystal structure: contains datablock(s) global, I. DOI: 10.1107/S1600536812005107/hg5175sup1.cif

Structure factors: contains datablock(s) I. DOI: 10.1107/S1600536812005107/hg5175Isup2.hkl

Supplementary material file. DOI: 10.1107/S1600536812005107/hg5175Isup3.cml

Additional supplementary materials:  crystallographic information; 3D view; checkCIF report

## Figures and Tables

**Table 1:** Hydrogen-bond geometry (Å, °)

*D*—H⋯*A*	*D*—H	H⋯*A*	*D*⋯*A*	*D*—H⋯*A*
O1—H1O⋯N6^i^	0.85 (1)	1.86 (1)	2.699 (3)	170 (5)
C13—H13*B*⋯S1^ii^	0.99	2.72	3.700 (2)	170
C22—H22⋯O1^iii^	0.95	2.33	3.149 (3)	145

Symmetry codes: (i) 
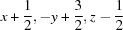
; (ii) 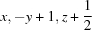; (iii) 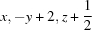.

**Fig. 1.**
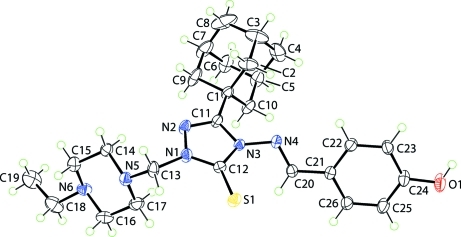
The molecular structure of (I) showing the atom-labelling scheme and displacement ellipsoids at the 50% probability level.

**Fig. 2.**
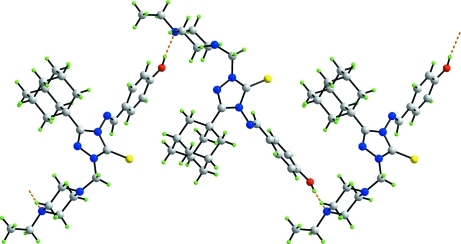
A view of the zigzag supramolecular chain along [100] in (I). The O—H···N hydrogen bonds are shown as orange dashed lines.

**Fig. 3.**
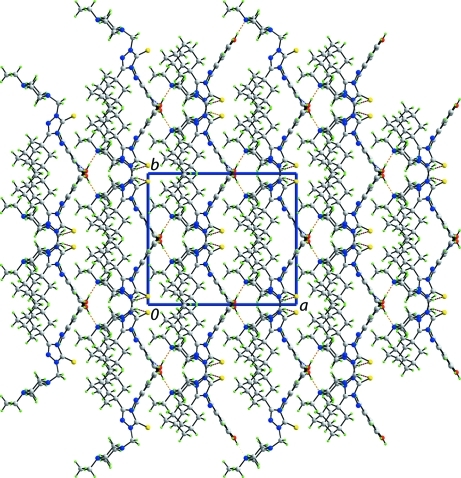
A view in projection down the c axis of the unit-cell contents for (I). The O—H···N, C—H···S and C—H···O interactions are shown as orange, brown and blue dashed lines, respectively.

## supplementary crystallographic information

### Comment

Derivatives of adamantane have long been known for their diverse biological activities including anti-viral activity against the influenza (Vernier *et al.*, 1969) and HIV viruses (El-Emam *et al.*, 2004). Moreover, adamantane derivative were recently reported to exhibit marked anti-bacterial and anti-inflammatory activities (Kadi *et al.*, 2007, 2010). In continuation of our interest in the chemical and pharmacological properties of adamantane derivatives, and as a part of on-going structural studies of adamantane derivatives (Kadi *et al.*, 2011; Al-Tamimi *et al.*, 2010) we synthesized the title compound (I) as potential chemotherapeutic agent.

The structure determination of (I), Fig. 1, confirms the presence of the thione in the solid-state. The central 1,2,4-triazole ring is planar [r.m.s. deviation = 0.020 Å] and the benzene ring is twisted out of this plane [dihedral angle = 62.35 (12)°]. Globally, the plane of the five-membered ring bisects the adamantyl group with the benzene and piperazine substituents lying to one side.

In the crystal packing, supramolecular zigzag chains are formed *via* O—H···N(piperazine) hydrogen bonds, Fig. 2 and Table 1. These are connected into the three-dimensional crystal structure by C—H···S and C—H···O interactions, Fig. 3 and Table 1.

### Experimental

A mixture of 709 mg (2 mmol) of 3-(1-adamantyl)-4-(4-hydroxybenzylideneamino)-4*H*-1,2,4-triazole-5-thiol (Al-Omar *et al.*, 2010), 1-ethylpiperazine (228 mg, 2 mmol) and 37% formaldehyde solution (1 ml), in ethanol (8 ml) was heated under reflux for 15 min when a clear solution was obtained. Stirring was continued for 12 h at room temperature and the mixture was allowed to stand overnight. Cold water (5 ml) was added and the mixture was stirred for 20 min. The precipitated crude product were filtered, washed with water, dried, and crystallized from ethanol to yield 425 mg (44%) of the title compound (I) as colourless crystals. m.p. 436–438 K. ^1^H NMR (CDCl_3_): δ 1.18 (t, 3H, CH_3_, J = 6.5 Hz), 1.69–1.76 (m, 6H, adamantane-H), 1.95 (s, 6H, adamantane-H), 2.03 (s, 3H, adamantane-H), 2.56 (q, 2H, CH_2_CH_3_, J = 6.5 Hz), 2.85–3.90 (m, 8H, piperazine-H), 5.18 (s, 2H, CH_2_), 6.75 (d, 2H, Ar—H, J = 8.0 Hz), 7.67 (d, 2H, Ar—H, J = 8.0 Hz), 9.21 (s, 1H, CH═N). ^13^C NMR: δ 11.11 (CH_3_), 27.84, 35.09, 36.48, 38.39 (adamantane-C), 49.50 (CH_2_CH_3_), 52.29, 58.43 (piperazine-C), 68.56 (CH_2_), 116.53, 123.49, 130.87, 161.53 (Ar—C), 154.94 (triazole C-3), 163.34 (CH═N), 164.65 (C═ S).

### Refinement

Carbon-bound H atoms were placed in calculated positions [C—H = 0.95 to 1.00 Å, *U*_iso_(H) = 1.2 to 1.5 *U*_eq_(C)] and were included in the refinement in the riding model approximation. The hydroxy H atom was located in a difference Fourier map, and was refined with a distance restraint of O—H = 0.84±0.01 Å, and *U*_iso_ was refined.

Owing to poor agreement a number of reflections, *i.e.* (11 1 4), (4 0 6), (8 4 2), (15 1 2), (10 2 1), (9 5 1), (13 1 1), (14 4 2), (13 3 2) and (14 2 1), were omitted from the final refinement.

The crystal is a racemic twin; the Flack parameter was explicitly refined.

There is a significant Hirshfeld rigid-bond alert for the C7—C8 bond. Their displacement factors are somewhat large, indicating some disorder. However, no model for the disorder was resolved.

### Crystal data

**Table:**

C_26_H_36_N_6_OS	*F*(000) = 1032
*M**_r_* = 480.67	*D*_x_ = 1.218 Mg m^−^^3^
Monoclinic, *C**c*	Mo *K*α radiation, λ = 0.71073 Å
Hall symbol: C -2yc	Cell parameters from 4991 reflections
*a* = 16.1006 (6) Å	θ = 2.5–27.5°
*b* = 14.2182 (5) Å	µ = 0.15 mm^−^^1^
*c* = 11.4641 (4) Å	*T* = 100 K
β = 92.440 (4)°	Prism, colourless
*V* = 2622.00 (16) Å^3^	0.25 × 0.20 × 0.15 mm
*Z* = 4	

### Data collection

**Table:**

Agilent SuperNova Dual diffractometer with Atlas detector	4840 independent reflections
Radiation source: SuperNova (Mo) X-ray Source	4374 reflections with *I* > 2σ(*I*)
Mirror monochromator	*R*_int_ = 0.036
Detector resolution: 10.4041 pixels mm^-1^	θ_max_ = 27.6°, θ_min_ = 2.5°
ω scans	*h* = −20→16
Absorption correction: multi-scan (*CrysAlis PRO*; Agilent, 2011)	*k* = −18→18
*T*_min_ = 0.963, *T*_max_ = 0.977	*l* = −14→14
12526 measured reflections	

### Refinement

**Table:**

Refinement on *F*^2^	Secondary atom site location: difference Fourier map
Least-squares matrix: full	Hydrogen site location: inferred from neighbouring sites
*R*[*F*^2^ > 2σ(*F*^2^)] = 0.039	H atoms treated by a mixture of independent and constrained refinement
*wR*(*F*^2^) = 0.100	*w* = 1/[σ^2^(*F*_o_^2^) + (0.0544*P*)^2^ + 0.5109*P*] where *P* = (*F*_o_^2^ + 2*F*_c_^2^)/3
*S* = 1.03	(Δ/σ)_max_ = 0.001
4840 reflections	Δρ_max_ = 0.25 e Å^−^^3^
312 parameters	Δρ_min_ = −0.18 e Å^−^^3^
3 restraints	Absolute structure: Flack (1983), with 1811 Friedel pairs
Primary atom site location: structure-invariant direct methods	Flack parameter: 0.06 (7)

### Fractional atomic coordinates and isotropic or equivalent isotropic displacement parameters (Å^2^)

**Table:**

	*x*	*y*	*z*	*U*_iso_*/*U*_eq_	
S1	0.49872 (4)	0.56121 (4)	0.50028 (4)	0.02603 (13)	
O1	0.57958 (13)	1.01838 (13)	0.12630 (14)	0.0397 (5)	
H1O	0.602 (3)	1.0657 (19)	0.160 (4)	0.103 (16)*	
N1	0.38211 (12)	0.55968 (12)	0.66504 (16)	0.0249 (4)	
N2	0.32689 (12)	0.61698 (13)	0.71956 (16)	0.0268 (4)	
N3	0.38731 (11)	0.69230 (12)	0.57892 (15)	0.0201 (4)	
N4	0.41828 (11)	0.77076 (12)	0.51938 (14)	0.0210 (4)	
N5	0.32453 (12)	0.40404 (13)	0.71060 (16)	0.0257 (4)	
N6	0.16089 (12)	0.32789 (13)	0.70910 (17)	0.0292 (4)	
C1	0.28270 (14)	0.78354 (15)	0.69923 (18)	0.0244 (5)	
C2	0.34125 (17)	0.85912 (18)	0.7529 (2)	0.0387 (6)	
H2A	0.3724	0.8331	0.8219	0.046*	
H2B	0.3818	0.8788	0.6952	0.046*	
C3	0.2896 (2)	0.9442 (2)	0.7892 (3)	0.0545 (9)	
H3	0.3275	0.9931	0.8245	0.065*	
C4	0.2445 (2)	0.98457 (18)	0.6805 (3)	0.0486 (8)	
H4A	0.2134	1.0418	0.7014	0.058*	
H4B	0.2853	1.0021	0.6221	0.058*	
C5	0.18436 (17)	0.91073 (17)	0.6291 (2)	0.0350 (6)	
H5	0.1535	0.9376	0.5593	0.042*	
C6	0.12260 (17)	0.88242 (19)	0.7202 (2)	0.0409 (6)	
H6A	0.0906	0.9382	0.7439	0.049*	
H6B	0.0829	0.8356	0.6864	0.049*	
C7	0.1689 (2)	0.8407 (2)	0.8263 (2)	0.0474 (8)	
H7	0.1281	0.8216	0.8853	0.057*	
C8	0.2276 (2)	0.9136 (3)	0.8790 (3)	0.0678 (12)	
H8A	0.2579	0.8870	0.9483	0.081*	
H8B	0.1956	0.9688	0.9045	0.081*	
C9	0.21912 (18)	0.75416 (19)	0.7898 (2)	0.0408 (7)	
H9A	0.1810	0.7059	0.7556	0.049*	
H9B	0.2486	0.7266	0.8592	0.049*	
C10	0.23419 (15)	0.82507 (16)	0.5923 (2)	0.0281 (5)	
H10A	0.2735	0.8437	0.5323	0.034*	
H10B	0.1961	0.7770	0.5578	0.034*	
C11	0.33174 (14)	0.69771 (15)	0.66707 (18)	0.0231 (4)	
C12	0.42254 (13)	0.60297 (14)	0.58011 (18)	0.0205 (4)	
C13	0.39656 (14)	0.46264 (15)	0.7065 (2)	0.0273 (5)	
H13A	0.4365	0.4323	0.6551	0.033*	
H13B	0.4232	0.4655	0.7859	0.033*	
C14	0.27018 (14)	0.42163 (15)	0.80734 (18)	0.0240 (5)	
H14A	0.2378	0.4799	0.7926	0.029*	
H14B	0.3039	0.4297	0.8809	0.029*	
C15	0.21187 (14)	0.33867 (16)	0.8179 (2)	0.0278 (5)	
H15A	0.2445	0.2806	0.8333	0.033*	
H15B	0.1754	0.3489	0.8841	0.033*	
C16	0.21480 (17)	0.31573 (17)	0.6094 (2)	0.0353 (6)	
H16A	0.1800	0.3122	0.5363	0.042*	
H16B	0.2457	0.2558	0.6184	0.042*	
C17	0.27601 (17)	0.39628 (17)	0.6011 (2)	0.0323 (5)	
H17A	0.3134	0.3847	0.5362	0.039*	
H17B	0.2456	0.4557	0.5850	0.039*	
C18	0.10554 (18)	0.24398 (19)	0.7131 (3)	0.0436 (7)	
H18A	0.1404	0.1868	0.7211	0.052*	
H18B	0.0729	0.2393	0.6381	0.052*	
C19	0.04676 (18)	0.2464 (2)	0.8111 (3)	0.0496 (7)	
H19A	0.0136	0.1886	0.8101	0.074*	
H19B	0.0098	0.3009	0.8014	0.074*	
H19C	0.0785	0.2514	0.8857	0.074*	
C20	0.42424 (13)	0.75687 (15)	0.40960 (18)	0.0211 (4)	
H20	0.4039	0.7001	0.3753	0.025*	
C21	0.46203 (13)	0.82785 (14)	0.33739 (18)	0.0213 (4)	
C22	0.50449 (14)	0.90456 (15)	0.38680 (19)	0.0250 (5)	
H22	0.5069	0.9122	0.4692	0.030*	
C23	0.54296 (15)	0.96938 (15)	0.31823 (19)	0.0267 (5)	
H23	0.5711	1.0216	0.3534	0.032*	
C24	0.54077 (15)	0.95855 (17)	0.19726 (19)	0.0284 (5)	
C25	0.49716 (16)	0.88341 (18)	0.1466 (2)	0.0325 (6)	
H25	0.4938	0.8768	0.0640	0.039*	
C26	0.45868 (15)	0.81838 (16)	0.21584 (18)	0.0275 (5)	
H26	0.4297	0.7668	0.1806	0.033*	

### Atomic displacement parameters (Å^2^)

**Table:**

	*U*^11^	*U*^22^	*U*^33^	*U*^12^	*U*^13^	*U*^23^
S1	0.0246 (3)	0.0275 (2)	0.0267 (3)	0.0066 (2)	0.0095 (2)	0.0022 (2)
O1	0.0465 (12)	0.0445 (10)	0.0276 (9)	−0.0220 (9)	−0.0034 (8)	0.0134 (8)
N1	0.0260 (10)	0.0223 (9)	0.0272 (9)	0.0052 (8)	0.0092 (8)	0.0052 (7)
N2	0.0282 (10)	0.0265 (9)	0.0266 (9)	0.0083 (8)	0.0129 (8)	0.0063 (8)
N3	0.0206 (9)	0.0206 (8)	0.0196 (8)	0.0011 (7)	0.0060 (7)	0.0006 (7)
N4	0.0205 (9)	0.0217 (8)	0.0212 (9)	−0.0005 (7)	0.0042 (7)	0.0046 (7)
N5	0.0257 (10)	0.0230 (9)	0.0288 (9)	0.0039 (8)	0.0056 (8)	0.0029 (8)
N6	0.0253 (11)	0.0304 (10)	0.0319 (10)	0.0012 (8)	0.0030 (8)	−0.0066 (8)
C1	0.0272 (12)	0.0259 (11)	0.0206 (10)	0.0077 (9)	0.0068 (9)	0.0033 (9)
C2	0.0395 (15)	0.0363 (13)	0.0396 (14)	0.0138 (12)	−0.0057 (12)	−0.0131 (12)
C3	0.0535 (19)	0.0411 (16)	0.068 (2)	0.0176 (14)	−0.0071 (17)	−0.0274 (15)
C4	0.0480 (18)	0.0238 (12)	0.075 (2)	0.0142 (12)	0.0164 (15)	0.0012 (13)
C5	0.0321 (14)	0.0366 (12)	0.0369 (14)	0.0157 (11)	0.0105 (11)	0.0127 (11)
C6	0.0364 (15)	0.0418 (14)	0.0459 (15)	0.0190 (12)	0.0185 (12)	0.0127 (12)
C7	0.0552 (19)	0.0547 (17)	0.0344 (14)	0.0306 (15)	0.0245 (13)	0.0088 (13)
C8	0.083 (3)	0.082 (2)	0.0383 (16)	0.057 (2)	0.0067 (17)	−0.0153 (16)
C9	0.0424 (16)	0.0485 (15)	0.0334 (13)	0.0243 (13)	0.0248 (12)	0.0166 (12)
C10	0.0256 (12)	0.0369 (12)	0.0223 (10)	0.0081 (10)	0.0054 (9)	0.0047 (10)
C11	0.0249 (11)	0.0261 (10)	0.0186 (10)	0.0036 (9)	0.0059 (8)	0.0043 (9)
C12	0.0196 (11)	0.0219 (10)	0.0200 (10)	0.0016 (8)	0.0005 (8)	−0.0004 (9)
C13	0.0249 (12)	0.0245 (10)	0.0332 (12)	0.0062 (9)	0.0083 (10)	0.0106 (10)
C14	0.0236 (12)	0.0257 (10)	0.0226 (10)	0.0018 (9)	0.0012 (9)	0.0021 (9)
C15	0.0223 (12)	0.0295 (11)	0.0318 (12)	0.0020 (9)	0.0035 (10)	0.0037 (10)
C16	0.0367 (15)	0.0345 (12)	0.0348 (13)	0.0057 (11)	0.0028 (11)	−0.0121 (11)
C17	0.0391 (15)	0.0321 (12)	0.0261 (11)	0.0082 (10)	0.0043 (10)	−0.0020 (10)
C18	0.0300 (15)	0.0367 (13)	0.0640 (18)	−0.0040 (11)	0.0005 (13)	−0.0110 (13)
C19	0.0297 (15)	0.0518 (16)	0.067 (2)	−0.0095 (13)	0.0024 (14)	−0.0083 (15)
C20	0.0181 (11)	0.0201 (9)	0.0252 (11)	0.0008 (8)	0.0032 (8)	0.0020 (8)
C21	0.0189 (11)	0.0234 (10)	0.0217 (10)	0.0012 (8)	0.0027 (8)	0.0042 (9)
C22	0.0260 (12)	0.0276 (10)	0.0216 (10)	−0.0014 (9)	0.0018 (9)	0.0015 (9)
C23	0.0282 (12)	0.0243 (10)	0.0274 (11)	−0.0080 (9)	0.0001 (10)	0.0027 (9)
C24	0.0262 (12)	0.0323 (12)	0.0265 (11)	−0.0065 (9)	−0.0002 (10)	0.0103 (9)
C25	0.0362 (15)	0.0405 (13)	0.0206 (10)	−0.0119 (11)	−0.0007 (10)	0.0054 (10)
C26	0.0286 (13)	0.0293 (11)	0.0246 (11)	−0.0078 (10)	0.0020 (10)	0.0002 (9)

### Geometric parameters (Å, º)

**Table:**

S1—C12	1.670 (2)	C7—H7	1.0000
O1—C24	1.349 (3)	C8—H8A	0.9900
O1—H1O	0.850 (10)	C8—H8B	0.9900
N1—C12	1.343 (3)	C9—H9A	0.9900
N1—N2	1.376 (3)	C9—H9B	0.9900
N1—C13	1.475 (3)	C10—H10A	0.9900
N2—C11	1.300 (3)	C10—H10B	0.9900
N3—C11	1.380 (3)	C13—H13A	0.9900
N3—C12	1.391 (3)	C13—H13B	0.9900
N3—N4	1.410 (2)	C14—C15	1.516 (3)
N4—C20	1.282 (3)	C14—H14A	0.9900
N5—C13	1.430 (3)	C14—H14B	0.9900
N5—C17	1.455 (3)	C15—H15A	0.9900
N5—C14	1.463 (3)	C15—H15B	0.9900
N6—C15	1.472 (3)	C16—C17	1.517 (4)
N6—C16	1.474 (3)	C16—H16A	0.9900
N6—C18	1.491 (3)	C16—H16B	0.9900
C1—C11	1.508 (3)	C17—H17A	0.9900
C1—C10	1.543 (3)	C17—H17B	0.9900
C1—C2	1.540 (3)	C18—C19	1.500 (4)
C1—C9	1.547 (3)	C18—H18A	0.9900
C2—C3	1.535 (3)	C18—H18B	0.9900
C2—H2A	0.9900	C19—H19A	0.9800
C2—H2B	0.9900	C19—H19B	0.9800
C3—C8	1.527 (6)	C19—H19C	0.9800
C3—C4	1.527 (4)	C20—C21	1.455 (3)
C3—H3	1.0000	C20—H20	0.9500
C4—C5	1.529 (4)	C21—C22	1.395 (3)
C4—H4A	0.9900	C21—C26	1.399 (3)
C4—H4B	0.9900	C22—C23	1.376 (3)
C5—C6	1.527 (4)	C22—H22	0.9500
C5—C10	1.528 (3)	C23—C24	1.394 (3)
C5—H5	1.0000	C23—H23	0.9500
C6—C7	1.519 (4)	C24—C25	1.392 (3)
C6—H6A	0.9900	C25—C26	1.382 (3)
C6—H6B	0.9900	C25—H25	0.9500
C7—C8	1.512 (5)	C26—H26	0.9500
C7—C9	1.540 (3)		
			
C24—O1—H1O	115 (3)	C5—C10—H10B	109.7
C12—N1—N2	113.61 (17)	C1—C10—H10B	109.7
C12—N1—C13	126.00 (18)	H10A—C10—H10B	108.2
N2—N1—C13	120.25 (18)	N2—C11—N3	110.08 (19)
C11—N2—N1	105.06 (18)	N2—C11—C1	124.0 (2)
C11—N3—C12	108.69 (17)	N3—C11—C1	125.92 (19)
C11—N3—N4	124.34 (18)	N1—C12—N3	102.45 (18)
C12—N3—N4	125.08 (18)	N1—C12—S1	128.61 (15)
C20—N4—N3	113.35 (17)	N3—C12—S1	128.90 (16)
C13—N5—C17	114.98 (19)	N5—C13—N1	116.01 (18)
C13—N5—C14	115.93 (18)	N5—C13—H13A	108.3
C17—N5—C14	110.53 (18)	N1—C13—H13A	108.3
C15—N6—C16	110.09 (18)	N5—C13—H13B	108.3
C15—N6—C18	111.71 (19)	N1—C13—H13B	108.3
C16—N6—C18	107.59 (19)	H13A—C13—H13B	107.4
C11—C1—C10	111.59 (18)	N5—C14—C15	108.67 (18)
C11—C1—C2	110.14 (19)	N5—C14—H14A	110.0
C10—C1—C2	109.34 (19)	C15—C14—H14A	110.0
C11—C1—C9	108.30 (18)	N5—C14—H14B	110.0
C10—C1—C9	108.06 (19)	C15—C14—H14B	110.0
C2—C1—C9	109.3 (2)	H14A—C14—H14B	108.3
C3—C2—C1	109.2 (2)	N6—C15—C14	109.78 (18)
C3—C2—H2A	109.8	N6—C15—H15A	109.7
C1—C2—H2A	109.8	C14—C15—H15A	109.7
C3—C2—H2B	109.8	N6—C15—H15B	109.7
C1—C2—H2B	109.8	C14—C15—H15B	109.7
H2A—C2—H2B	108.3	H15A—C15—H15B	108.2
C8—C3—C4	110.8 (3)	N6—C16—C17	111.47 (19)
C8—C3—C2	109.6 (3)	N6—C16—H16A	109.3
C4—C3—C2	108.6 (2)	C17—C16—H16A	109.3
C8—C3—H3	109.3	N6—C16—H16B	109.3
C4—C3—H3	109.3	C17—C16—H16B	109.3
C2—C3—H3	109.3	H16A—C16—H16B	108.0
C5—C4—C3	109.1 (2)	N5—C17—C16	109.2 (2)
C5—C4—H4A	109.9	N5—C17—H17A	109.8
C3—C4—H4A	109.9	C16—C17—H17A	109.8
C5—C4—H4B	109.9	N5—C17—H17B	109.8
C3—C4—H4B	109.9	C16—C17—H17B	109.8
H4A—C4—H4B	108.3	H17A—C17—H17B	108.3
C6—C5—C10	110.1 (2)	N6—C18—C19	113.7 (2)
C6—C5—C4	109.7 (2)	N6—C18—H18A	108.8
C10—C5—C4	108.8 (2)	C19—C18—H18A	108.8
C6—C5—H5	109.4	N6—C18—H18B	108.8
C10—C5—H5	109.4	C19—C18—H18B	108.8
C4—C5—H5	109.4	H18A—C18—H18B	107.7
C7—C6—C5	109.8 (2)	C18—C19—H19A	109.5
C7—C6—H6A	109.7	C18—C19—H19B	109.5
C5—C6—H6A	109.7	H19A—C19—H19B	109.5
C7—C6—H6B	109.7	C18—C19—H19C	109.5
C5—C6—H6B	109.7	H19A—C19—H19C	109.5
H6A—C6—H6B	108.2	H19B—C19—H19C	109.5
C8—C7—C6	109.2 (3)	N4—C20—C21	120.16 (19)
C8—C7—C9	109.3 (3)	N4—C20—H20	119.9
C6—C7—C9	109.9 (2)	C21—C20—H20	119.9
C8—C7—H7	109.5	C22—C21—C26	118.51 (19)
C6—C7—H7	109.5	C22—C21—C20	121.40 (19)
C9—C7—H7	109.5	C26—C21—C20	120.05 (19)
C7—C8—C3	109.9 (2)	C23—C22—C21	121.1 (2)
C7—C8—H8A	109.7	C23—C22—H22	119.4
C3—C8—H8A	109.7	C21—C22—H22	119.4
C7—C8—H8B	109.7	C22—C23—C24	120.1 (2)
C3—C8—H8B	109.7	C22—C23—H23	120.0
H8A—C8—H8B	108.2	C24—C23—H23	120.0
C7—C9—C1	109.7 (2)	O1—C24—C25	118.1 (2)
C7—C9—H9A	109.7	O1—C24—C23	122.5 (2)
C1—C9—H9A	109.7	C25—C24—C23	119.4 (2)
C7—C9—H9B	109.7	C26—C25—C24	120.3 (2)
C1—C9—H9B	109.7	C26—C25—H25	119.9
H9A—C9—H9B	108.2	C24—C25—H25	119.9
C5—C10—C1	109.84 (19)	C25—C26—C21	120.6 (2)
C5—C10—H10A	109.7	C25—C26—H26	119.7
C1—C10—H10A	109.7	C21—C26—H26	119.7
			
C12—N1—N2—C11	0.8 (2)	C10—C1—C11—N3	52.9 (3)
C13—N1—N2—C11	176.73 (19)	C2—C1—C11—N3	−68.8 (3)
C11—N3—N4—C20	−140.2 (2)	C9—C1—C11—N3	171.7 (2)
C12—N3—N4—C20	57.3 (3)	N2—N1—C12—N3	−2.5 (2)
C11—C1—C2—C3	−177.9 (2)	C13—N1—C12—N3	−178.14 (19)
C10—C1—C2—C3	59.2 (3)	N2—N1—C12—S1	175.18 (16)
C9—C1—C2—C3	−59.0 (3)	C13—N1—C12—S1	−0.5 (3)
C1—C2—C3—C8	60.0 (3)	C11—N3—C12—N1	3.2 (2)
C1—C2—C3—C4	−61.2 (3)	N4—N3—C12—N1	168.07 (18)
C8—C3—C4—C5	−57.6 (3)	C11—N3—C12—S1	−174.48 (16)
C2—C3—C4—C5	62.8 (3)	N4—N3—C12—S1	−9.6 (3)
C3—C4—C5—C6	58.3 (3)	C17—N5—C13—N1	55.3 (3)
C3—C4—C5—C10	−62.2 (3)	C14—N5—C13—N1	−75.8 (2)
C10—C5—C6—C7	59.2 (3)	C12—N1—C13—N5	−128.4 (2)
C4—C5—C6—C7	−60.5 (3)	N2—N1—C13—N5	56.2 (3)
C5—C6—C7—C8	60.8 (3)	C13—N5—C14—C15	−164.70 (17)
C5—C6—C7—C9	−59.0 (3)	C17—N5—C14—C15	62.2 (2)
C6—C7—C8—C3	−59.5 (3)	C16—N6—C15—C14	57.6 (2)
C9—C7—C8—C3	60.8 (3)	C18—N6—C15—C14	177.0 (2)
C4—C3—C8—C7	58.6 (3)	N5—C14—C15—N6	−60.5 (2)
C2—C3—C8—C7	−61.2 (3)	C15—N6—C16—C17	−55.8 (2)
C8—C7—C9—C1	−59.7 (3)	C18—N6—C16—C17	−177.7 (2)
C6—C7—C9—C1	60.1 (3)	C13—N5—C17—C16	166.65 (19)
C11—C1—C9—C7	179.0 (2)	C14—N5—C17—C16	−59.7 (2)
C10—C1—C9—C7	−59.9 (3)	N6—C16—C17—N5	56.4 (2)
C2—C1—C9—C7	59.0 (3)	C15—N6—C18—C19	59.0 (3)
C6—C5—C10—C1	−60.2 (3)	C16—N6—C18—C19	179.9 (2)
C4—C5—C10—C1	60.1 (3)	N3—N4—C20—C21	−174.54 (17)
C11—C1—C10—C5	179.1 (2)	N4—C20—C21—C22	10.6 (3)
C2—C1—C10—C5	−58.8 (3)	N4—C20—C21—C26	−171.5 (2)
C9—C1—C10—C5	60.1 (3)	C26—C21—C22—C23	−0.5 (3)
N1—N2—C11—N3	1.3 (2)	C20—C21—C22—C23	177.3 (2)
N1—N2—C11—C1	−179.0 (2)	C21—C22—C23—C24	−0.7 (4)
C12—N3—C11—N2	−3.0 (2)	C22—C23—C24—O1	−177.9 (2)
N4—N3—C11—N2	−167.98 (19)	C22—C23—C24—C25	2.0 (4)
C12—N3—C11—C1	177.3 (2)	O1—C24—C25—C26	177.8 (2)
N4—N3—C11—C1	12.3 (3)	C23—C24—C25—C26	−2.1 (4)
C10—C1—C11—N2	−126.8 (2)	C24—C25—C26—C21	0.9 (4)
C2—C1—C11—N2	111.6 (3)	C22—C21—C26—C25	0.4 (3)
C9—C1—C11—N2	−8.0 (3)	C20—C21—C26—C25	−177.5 (2)

### Hydrogen-bond geometry (Å, º)

**Table:**

*D*—H···*A*	*D*—H	H···*A*	*D*···*A*	*D*—H···*A*
O1—H1*O*···N6^i^	0.85 (1)	1.86 (1)	2.699 (3)	170 (5)
C13—H13*B*···S1^ii^	0.99	2.72	3.700 (2)	170
C22—H22···O1^iii^	0.95	2.33	3.149 (3)	145

Symmetry codes: (i) *x*+1/2, −*y*+3/2, *z*−1/2; (ii) *x*, −*y*+1, *z*+1/2; (iii) *x*, −*y*+2, *z*+1/2.
